# Adherence to the Mediterranean Diet Mitigates Inflammation and Hospital Stay in Frail Elderly Patients: A Moderation Analysis

**DOI:** 10.3390/nu16152482

**Published:** 2024-07-31

**Authors:** Aurelio Lo Buglio, Francesco Bellanti, Rosanna Maria Carapellese, Cristiano Capurso, Gaetano Serviddio, Gianluigi Vendemiale

**Affiliations:** Department of Medical and Surgical Sciences, University of Foggia, Viale Pinto 1, 71122 Foggia, Italy; aurelio.lobuglio@unifg.it (A.L.B.); rosanna_carapellese.549375@unifg.it (R.M.C.); cristiano.capurso@unifg.it (C.C.); gaetano.serviddio@unifg.it (G.S.); gianluigi.vendemiale@unifg.it (G.V.)

**Keywords:** malnutrition, elderly people, hospitalized elderly people, Mediterranean diet, inflammatory markers, CRP, length of stay, frailty, nutritional status

## Abstract

Understanding the interaction between dietary patterns and nutritional status in influencing health outcomes is crucial, especially in vulnerable populations. Our study investigates the impact of adherence to the Mediterranean diet (MD) and nutritional status on inflammatory markers (CRP) and the length of stay (LOS) in hospitalized frail elderly patients. Methods: We conducted two-way ANOVA and multiple regression analysis to evaluate the effects of nutritional status and MD adherence on the CRP levels and LOS in a cohort of 117 frail elderly patients aged 65 years or older. Patients with cancer or acute infection were excluded. Adherence to the MD was assessed using the 14-item PREDIMED questionnaire. Results: Significant interactions were found between nutritional status and MD adherence for both the CRP and LOS. The patients with low-level MD adherence and a poor nutritional status exhibited higher CRP levels and longer hospital stays compared to those with high MD adherence. Specifically, a statistically significant interaction was observed for the CRP (*F* (1, 113) = 7.36, *p* = 0.008) and LOS (*F* (1, 113) = 15.4, *p* < 0.001), indicating the protective effect of high-level MD adherence. Moderation analysis confirmed that high-level MD adherence mitigates the adverse effects of malnutrition on both the inflammatory response and LOS. Conclusions: These findings highlight the importance of promoting the MD, particularly in malnourished elderly patients, to improve health outcomes and reduce hospitalization duration. Further longitudinal studies are warranted to establish causality and explore the underlying mechanisms.

## 1. Introduction

Malnutrition poses a significant challenge among elderly populations, especially those who are hospitalized [1,2]. The data indicate that about 25% of individuals aged 65 and older are either malnourished or at risk of malnutrition [3]. This issue becomes even more pronounced in hospitalized elderly patients, where the prevalence of malnutrition can exceed 70%, particularly among frail older adults [4]. The adverse outcomes associated with malnutrition in elderly people are extensive, including longer hospital stays, increased infection rates, higher chances of re-hospitalization, and elevated mortality rates [5,6,7]. Furthermore, a poor nutritional status affects muscle architecture and mass and impacts muscle performances [8,9]. The critical link between a poor nutritional status, frailty, and clinical outcomes is chronic low-grade inflammation [10,11,12]. In the absence of acute infection, malnutrition can induce and worsen the pro-inflammatory state, and chronic inflammation, in turn, can contribute to the onset or progression of malnutrition through various mechanisms involving numerous inflammatory cytokines [13,14]. Inflammation can lead to reduced appetite and food intake, worsening the nutritional status, while malnutrition can further aggravate inflammation. This reciprocal relationship can leads to worse clinical outcomes and extended hospital stays [13,15]. Furthermore, as well as acute illness, inflammation appears to be a predictive marker of clinical outcomes, including mortality, up to 24 months after hospital discharge [15,16]. Malnutrition is also associated with an increase in reactive oxygen species, and alterations in the redox balance play a crucial role in determining immune function and inflammatory responses [17,18].

Frail elderly patients are especially susceptible to the detrimental effects of malnutrition and inflammation [10,19]. Frailty, a syndrome marked by reduced strength, endurance, and physiological function, significantly raises the risk of adverse health outcomes, such as falls, disability, hospitalization, and death [20,21]. In the hospital setting, the interaction between malnutrition and inflammation is particularly severe in frail elderly patients [22]. Chronic low-grade inflammation plays a substantial role in the decline of functional status and exacerbates malnutrition [23]. Inflammatory cytokines such as interleukin-6 (IL-6) and tumor necrosis factor-alpha (TNF-α) are central to this process, contributing to muscle mass decline, increased fat mass, and overall frailty [24,25,26].

The MD, which emphasizes the high-level consumption of vegetables, fruits, whole grains, legumes, nuts, and olive oil, with the moderate consumption of fish and poultry, has been widely recognized for its health benefits [27]. The MD is associated with reduced mortality from cardiovascular diseases and protective effects against obesity, diabetes, neurodegenerative diseases, and certain cancers [28,29]. High-level adherence to the MD has demonstrated beneficial effects on lifespan, with up to a 25% reduction in total mortality [30]. The health benefits of the MD are believed to be due to its anti-inflammatory, antioxidant, and metabolic effects [31,32]. High-level adherence to the MD is also associated with a better nutritional status [6,33].

Individuals adhering to the MD tend to have a better body composition characterized by increased lean mass and decreased fat mass, which are crucial for maintaining physical functions and reducing frailty [34,35]. However, despite these benefits, adherence to the MD is declining, even in Mediterranean countries [36].

In the context of hospitalized elderly patients, especially those who are frail, adherence to the MD could significantly influence the impact of nutritional status on clinical outcomes. Research has demonstrated that higher adherence to the MD is linked to lower levels of systemic inflammation, as evidenced by markers like IL-6 and TNF-α [37].

This study aims to investigate the role of the MD in modulating the effects of nutritional status on inflammation and hospital stay duration in a population of frail elderly patients.

## 2. Materials and Methods

### 2.1. Study Design and Population

This is a retrospective single-center study conducted at the department of “Medicina Interna e dell’Invecchiamento”, “Policlinico Riuniti”, in Foggia (Italy). We analyzed the clinical data from 117 frailty patients, aged 65 years or older, who were admitted to our department between December 2023 and May 2024. The exclusion criteria included having active cancer, acute infection, or sepsis. Additionally, we excluded participants with missing data on nutritional status or on adherence to the MD.

### 2.2. Biochemical Analysis and Data Collection

At the time of hospital admission, a blood sample was collected for the assessment of the base clinical parameters, including hemoglobin, total white blood cell count (WBC), neutrophil count, lymphocyte count, platelet count, glucose, albumin, creatinine, total cholesterol, low-density lipoprotein (LDL), high-density lipoprotein (HDL), triglycerides, ferritin, and C-reactive protein (CRP). Body Mass Index (BMI) was also calculated by dividing the patient’s weight in kilograms by the square of their height in meters. The presence of at least three of the following conditions—hypertension, diabetes, chronic kidney disease (CKD, defined as an estimated Glomerular Filtration Rate below 60 mL/min/1.73 m^2^), heart failure (HF), ischemic chronic heart disease (ICHD), atrial fibrillation (AF), stroke, and chronic obstructive pulmonary disease (COPD)—was defined as the presence of comorbidities [38].

### 2.3. Assessment of Nutritional Status and Adherence to the Mediterranean Diet

The mini nutritional assessment (MNA) is a validated instrument designed to evaluate the nutritional status of hospitalized elderly patients. It includes 18 questions that cover anthropometric measurements, general health, dietary intake, and subjective evaluations. Based on the total score, the patients are categorized into one of three groups: malnourished (score below 17), at risk of malnutrition (score between 17 and 23.5), or well-nourished (score above 23.5) [39,40].

Adherence to the MD was assessed using the validated 14-item PREDIMED questionnaire [41]. This questionnaire was administered in person by a qualified nutritionist upon the patients’ admission. Each item on the questionnaire was scored either 1 or 0, resulting in a total possible score ranging from 0 to 14. Scores below 10 were classified as indicating low-level adherence to the MD, while scores of 10 or higher indicated high-level adherence [41,42].

### 2.4. Frailty Assessment

Frailty was defined according to Fried’s criteria as follows [43]:

1. Weight Loss: The unintentional loss of more than 4.5 kg in the last year or a ≥5% loss of body weight in the previous year.
2. Exhaustion: Measured using the CES-D Depression Scale, identified by the answers, “I felt that everything I did was an effort” and “I could not get going”.
3. Physical Activity: Evaluated using the short version of the Minnesota Leisure Time Activity questionnaire, with a weighted physical activity score calculated for each gender and kilocalories expended per week determined.
4. Walking Time: Based on the time required to walk 4.57 m (15 feet), adjusted for gender and standing height.
5. Grip Strength: Stratified by gender and BMI quartiles.

The patients were classified as frail if three or more indicators were present, pre-frail if one or two indicators were present, and robust if no indicators were present.

### 2.5. Statistical Analysis

The data are reported as counts and percentages for the qualitative variables and as mean ± standard deviation (SD) or median [interquartile range] for the quantitative variables. The normal distribution of the samples was assessed using the Kolmogorov–Smirnov test. Group comparisons for continuous variables were conducted using the *t*-test or the Mann–Whitney test for parametric or non-parametric distributions, respectively. Nominal and categorical variables were analyzed using Pearson’s Chi-Squared test.

To satisfy the assumptions of normality for two-way analysis of variance (ANOVA) and moderation analysis, the CRP levels and LOS were transformed using the natural logarithm (Ln). This transformation was necessary to correct for skewed distributions and to stabilize variance. Two-way ANOVA was performed to evaluate the effects of nutritional status assessed using the MNA and adherence to the MD on the circulating CRP levels and LOS. The Tukey test was employed for multiple comparisons test. Moderation analysis was conducted using the PROCESS macro in SPSS (written by Andrew F. Hayes) macro running Moderator-Model 1 to explore the moderating effect of adherence to the MD on the relationship between nutritional status and both the CRP levels and LOS. Analysis was adjusted for confounders: age, gender, and comorbidities. The interaction effects between adherence to the MD and nutritional status on CRP and LOS were assessed using F-tests. Main effects for nutritional status and adherence to the MD were also analyzed.

Power analysis was performed using G*Power software (version 3.1.9.7). For the ANOVA test, we used an effect size of *f* = 0.30, a significance level of α = 0.05, and a desired power of 0.80. The calculations indicated that a total sample size of 90 participants was necessary to detect moderate effects. Our study included 117 participants, achieving an actual power of 0.90, with a critical *F*-value of 3.923.

For moderation analysis, we considered an effect size of *f* = 0.15, a significance level of α = 0.05, and a desired power of 0.80. The calculations indicated that a total sample size of 85 participants was necessary to detect moderate effects. With our sample of 117 participants, we achieved an actual power of 0.926.

Statistical significance was defined as *p* < 0.05. All statistical analyses were conducted and graphs were created using Posit Software, version 2023.9.1.494 (PBC, Boston, MA, USA, URL: http://www.posit.co/). Moderation analysis was performed using the PROCESS macro in SPSS, version 22.0 (SPSS Inc., Chicago, IL, USA).

## 3. Results

### 3.1. Characteristics of the Population

**Figure 1:**
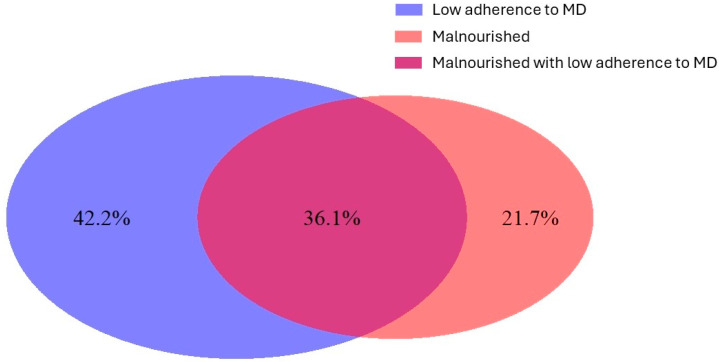
Venn diagram illustrating the overlap between the malnourished patients and the patients with low-level adherence to the Mediterranean diet. The percentages refer to the proportion of patients within these two specific conditions, not the entire population, which also includes the well-nourished patients and those with high-level adherence to the MD.

This study is an observational, non-randomized study. A total of 117 patients were enrolled retrospectively, of which 78 (66.7%) were female. The mean age was 79.3 ± 6.5 years. The reasons for hospital admission are reported in Table S1 in the Supplementary Materials. The level of adherence to the Mediterranean diet was high in 52 (44.4%) patients, while the remaining study population showed low-level adherence. Based on the MNA, 47 (40.2%) patients were malnourished, 50 (42.7%) were at risk of malnutrition, and 20 (17.1%) were well nourished. Considering the malnourished patients and patients with low-level adherence to the Mediterranean diet, the overlap of these two conditions is illustrated in a Venn diagram (Figure 1). The patients were divided into two groups according to adherence to the MD: high-level adherence and low-level adherence. The baseline characteristics are reported in Table 1. Approval for this research was obtained from the Institutional Review Board at Policlinico Foggia, and the study was carried out in line with the principles outlined in the Declaration of Helsinki.

The group with low-level adherence to the MD showed a significantly lower prevalence of females compared to that of the group with higher-level adherence. Additionally, the low-level adherence group had a higher prevalence of comorbidities and elevated levels of hemoglobin, triglycerides, creatinine, ferritin, and CRP. They also had lower levels of platelets, albumin, and HDL cholesterol. The patients with poor adherence to the MD experienced the longest LOS. The data are reported in Table 1.

### 3.2. Two-Way ANOVA Analysis: Impact of Nutritional Status and Mediterranean Diet Adherence

Two-way between-group ANOVA was conducted to evaluate the impact of nutritional status assessed using the MNA and adherence to the MD on the circulating CRP levels. Second analysis was also performed to examine the effect of these variables on the LOS.

**Figure 2:**
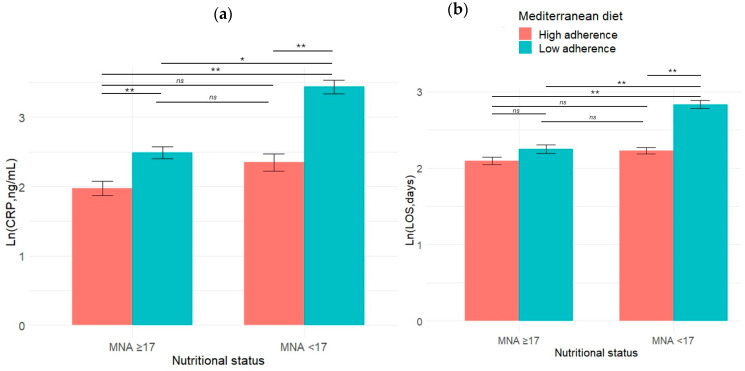
The differences between the groups in the mean values of (**a**) circulant levels of CRP expressed as Ln (CRP) and (**b**) LOS expressed as Ln (LOS). The bars in the graph represent the mean values of Ln (CRP, ng/mL) for each group, while the error bars indicate the standard deviation. Abbreviation: MNA, mini nutritional assessment; CRP, C reactive protein; LOS, length of stay; Ln (LOS), natural logarithm of length of stay. * *p* < 0.05; ** *p* < 0.001, ns, not significant.

**Figure 3:**
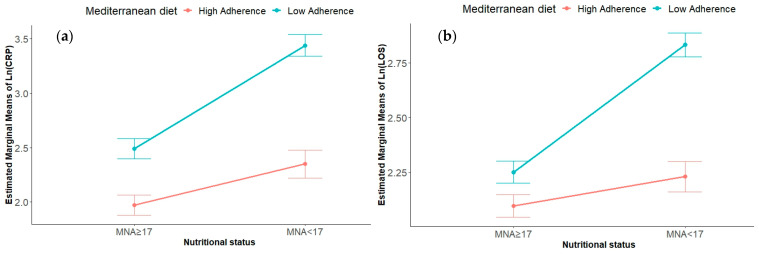
Interaction effects of nutritional status and adherence to the MD on (**a**) circulating CRP levels expressed as Ln (CRP) and (**b**) LOS expressed as Ln (CRP). Abbreviation: MNA, mini nutritional assessment; CRP, C reactive protein; LOS, length of stay; Ln (LOS), natural logarithm of length of stay.

The interaction effect between adherence to the MD and nutritional status on Ln (CRP) levels was statistically significant, *F* (1, 113) = 7.36, *p* = 0.008, with a medium effect size (partial eta squared = 0.06). The simple main effect analysis of nutritional status was statistically significant, *F* (1, 113) = 39.7, *p* < 0.001, indicating a large effect size (partial eta squared = 0.26). Similarly, the simple main effect of adherence to the MD was statistically significant, *F* (1, 113) = 58.4, *p* < 0.001, with a large effect size (partial eta squared = 0.34). The descriptive statistics are reported in Table 2. Statistically significant differences between the groups are reported in Figure 2a. A graphical representation of the interaction is reported in Figure 3a.

The interaction effect between adherence to the MD and nutritional status on Ln (LOS) was statistically significant, *F* (1, 113) = 15.4, *p* < 0.001, with a medium–large effect size (partial eta squared = 0.12). Simple main effect analysis for nutritional status was also statistically significant, *F* (1, 113) = 39.5, *p* < 0.001, indicating a large effect size (partial eta squared = 0.26). Additionally, the main effect of adherence to the MD was statistically significant, *F* (1, 113) = 44.2, *p* < 0.001, with a large effect size (partial eta squared = 0.28).

The descriptive statistics are reported in Table 2. The statistically significant differences between the groups are reported in Figure 2a. A graphical representation of the interaction is reported in Figure 3b.

### 3.3. Moderation Analysis

**Figure 4:**
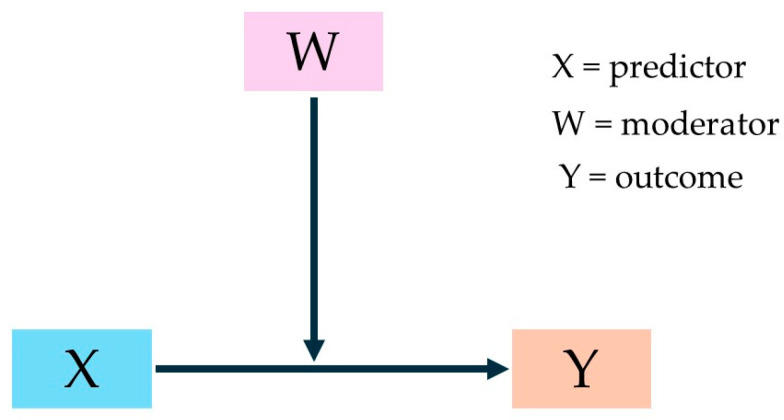
Simple moderation analysis.

Moderation analysis explores how the relationship between two variables, the predictor (X) and the outcome (Y), is influenced by a third variable known as the moderator (W). A moderator alters the strength or direction of this relationship, highlighting the conditions under which the relationship is enhanced, weakened, or changed in direction. Moderation is assessed through the interaction effect between the predictor variable and the moderator. A significant interaction effect suggests moderation, indicating that the relationship between the predictor and outcome is conditional on the moderator. The moderation model used in our study is represented in the Figure 4.

To analyze the moderating effect of adherence to the MD on nutritional and inflammatory statuses, the PROCESS macro for SPSS was used. Separate analysis was conducted to explore the impact of adherence to the MD on nutritional status and the length of stay.

**Figure 5:**
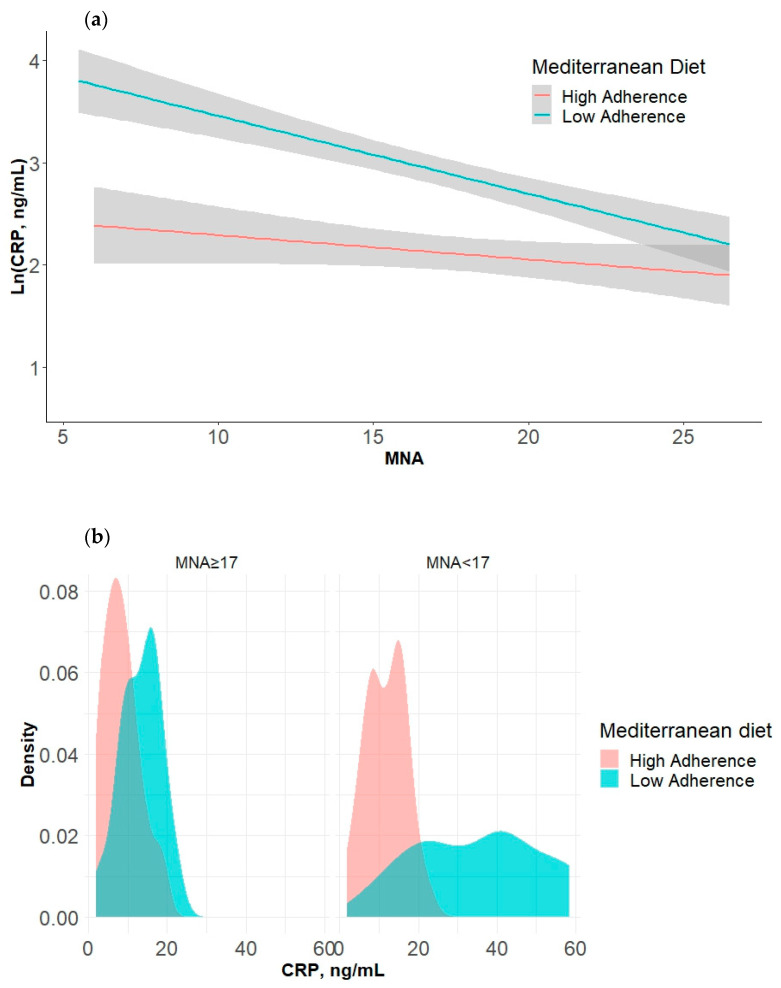
(**a**) An interaction plot illustrating the moderating effect of adherence to the Mediterranean diet on the relationship between nutritional status and Ln (CRP) levels. The area in grey is represented 95%CI. (**b**) A density plot illustrating the distribution of CRP levels across the different categories of Mediterranean diet adherence (low vs. high) and nutritional status. Each subplot represents a different MNA group, with the *x*-axis showing the CRP levels and the colors indicating Mediterranean diet adherence. Abbreviations: MNA, mini nutritional assessment; CRP, C-reactive protein; Ln (CRP), natural logarithm of CRP.

In the first analysis, a statistically significant interaction was observed (*F* (3, 113) = 33.41, *p* < 0.001, R squared = 0.47). The interaction between nutritional status and adherence to the MD significantly improved the model’s ability to explain the variance in inflammatory status (*F* (1, 113) = 7.94, *p* 0.06, R squared change = 0.04). There was no significant relationship found between nutritional status and inflammatory status when the level of adherence to the MD was low. However, with high-level adherence to the MD, a significantly negative relationship was identified between nutritional status and inflammatory status (*b* = −0.08, 95%CI [−0.10, −0.05], *t* = −6.10, *p* < 0.001). The results of multiple regression analysis are summarized in Table 3. The interaction is graphically represented in Figure 5.

**Figure 6:**
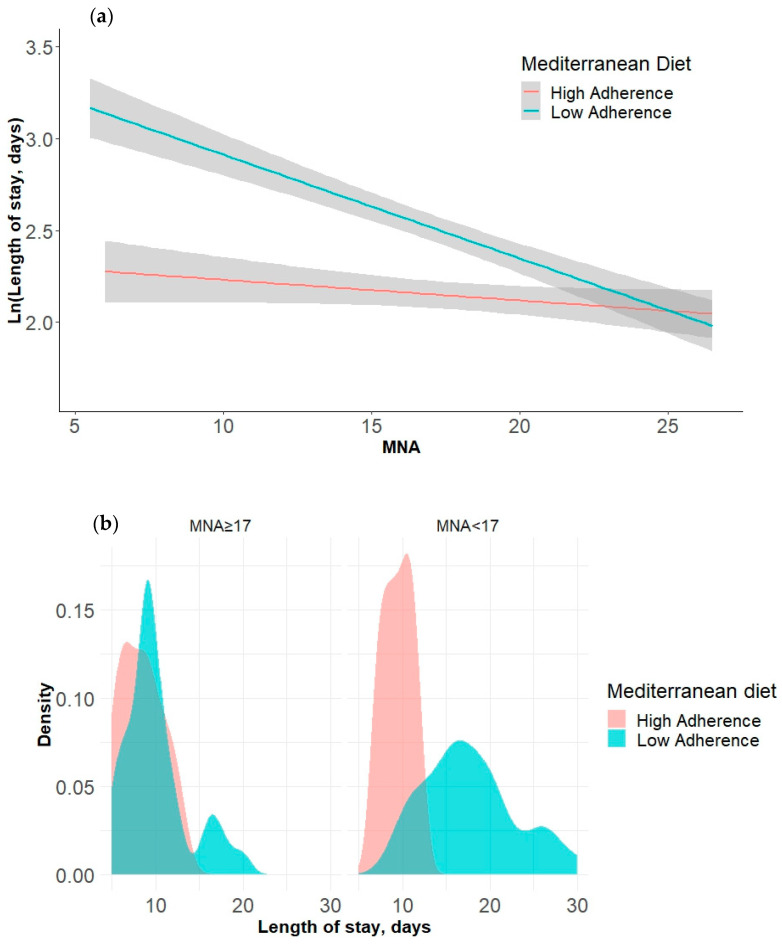
(**a**) An interaction plot illustrating the moderating effect of adherence to the Mediterranean diet on the relationship between nutritional status and Ln (LOS). The area in grey is represented 95%CI. (**b**) A density plot illustrating the distribution of length of stay across different categories of Mediterranean diet adherence (low vs. high) and nutritional status. Each subplot represents a different MNA group, with the *x*-axis showing the LOS and the colors indicating Mediterranean diet adherence. Abbreviations: MNA, mini nutritional assessment; LOS, length of stay; Ln (LOS), natural logarithm of LOS.

In the second analysis, we examined the moderating effect of adherence to the Mediterranean diet on the relationship between nutritional status and length of stay, revealing a significant interaction: *F* (3, 113) = 47.85, *p* < 0.001, R squared = 0.56. The interaction effect between nutritional status and adherence to the MD substantially enhanced the model’s capacity to account for the variability in length of stay: *F* (1, 113) = 24.21, *p* < 0.001, with a R square change of 0.10. No significant relationship was observed between nutritional status and length of stay when the level of adherence to the MD was low. However, with high-level adherence to the MD, a notably negative relationship emerged between nutritional status and length of stay, with a coefficient of *b* = −0.06, 95%CI [−0.07, −0.04], *t* = −9.35, *p* < 0.001. The results of the multiple regression analysis are summarized in Table 3. The interaction is graphically represented in Figure 6.

## 4. Discussion

This study highlights the significant interactions between adherence to the MD, nutritional status, inflammatory status (CRP), and LOS in hospitalized frail elderly patients, contributing novel insights to the literature.

Our findings reveal higher CRP levels among the patients with low-level adherence to the MD, underscoring the role of dietary patterns in influencing inflammatory markers in frail elderly populations. This aligns with the concept of “inflammaging” and the well-established link between malnutrition and heightened inflammatory responses [44,45,46]. Notably, in the presence of elevated MNA scores, no differences were observed between the patients with high- or low-level adherence to the MD. However, the malnourished patients with low-level MD adherence exhibited significantly higher CRP levels compared to those with high-level adherence (Figure 2a). Two-way ANOVA identified a significant interaction between nutritional status and MD adherence on the inflammatory response, emphasizing how these factors jointly influence health outcomes. Specifically, Figure 3a illustrates that both low MNA scores and low-level MD adherence showed higher CRP levels, a clinically relevant finding given the prevalence of concurrent conditions in more than 25% of our study cohort. Previous research has consistently shown that adherence to the MD is associated with lower inflammatory markers in various patient populations, including elderly people, cancer patients, and those with chronic diseases [6,47,48,49]. Our study extends this understanding to frail elderly patients, demonstrating a protective effect of high-level MD adherence against inflammatory responses exacerbated by malnutrition. Moderation analysis further elucidated the role of MD adherence in mitigating the impact of malnutrition on inflammation. A statistically significant interaction was observed, *F* (3, 113) = 33.41, *p* < 0.001, R squared = 0.47. The interaction between nutritional status and adherence to the MD significantly improved the model’s ability to explain variance of the inflammatory status, *F* (1, 113) = 7.94, *p* = 0.006, R squared change = 4%. There was no significant relationship found between nutritional status and inflammatory status when the level of adherence to the MD was low. However, with high-level adherence to the MD, a significantly negative relationship was identified between nutritional status and inflammatory status, *b* = −0.08, 95%CI [−0.10, −0.05], *t* = −6.10, *p* < 0.001. This highlights the specific components of the MD that contribute to its anti-inflammatory effects, such as its antioxidant-rich composition and a high content of monounsaturated and polyunsaturated fatty acids [50,51,52,53,54,55].

When we consider LOS as dependent variable, our results showed that the patients with low-level adherence to the MD experienced longer hospital stays compared to those with higher-level adherence, highlighting the potential impact of dietary patterns on LOS in frail elderly populations. Two-way ANOVA identified a significant interaction between nutritional status and MD adherence on LOS, as depicted in Figure 3b. Specifically, the patients characterized by both a poor nutritional status and low-level MD adherence exhibited a longer LOS compared to that of the other groups (Figure 2b).

We examined the moderating effect of adherence to the MD on the relationship between nutritional status and LOS, revealing a significant interaction: *F* (3, 113) = 47.85, *p* < 0.001, R squared = 0.56. The interaction effect between nutritional status and adherence to the MD substantially enhanced the model’s capacity to account for variability in length of stay: *F* (1, 113) = 24.21, *p* < 0.001, with an R squared change of 10%. No significant relationship was observed between nutritional status and length of stay when the level of adherence to the MD was low. However, with high-level adherence to the MD, a notably negative relationship emerged between nutritional status and length of stay, with a coefficient of *b* = −0.06, 95%CI [−0.07, −0.04], *t* = −9.35, *p* < 0.001. This underscores the importance of dietary interventions in mitigating adverse health outcomes, including extended hospital stays, among frail elderly patients [4,56]. This highlights specific components of the MD, such as its antioxidant-rich composition and healthy fats, which contribute to its potential to reduce LOS by promoting better immune function and overall metabolic health. Furthermore, the existing literature supports the notion that dietary interventions emphasizing antioxidants and healthy fats, as found in the MD, are associated with improved health outcomes and a potentially shorter LOS in various patient populations [57,58].

The present study had some limitations. First, the retrospective and cross-sectional design limited our ability to establish causality between adherence to the MD, nutritional status, inflammatory markers (CRP), and LOS. Longitudinal studies would provide more robust evidence of these relationship. Second, the study was conducted at a single center with a small simple size, which may limit the variability of hospital practices and patient demographics. Multi-center studies would provide more diverse perspectives and enhance the robustness of the findings. Third, although efforts were made to control for potential confounding variables, such as comorbidities, residual confounding may still exist. Other unmeasured variables, such as medication use, socioeconomic status, or physical activity levels, could influence the observed associations. Lastly, dietary adherence to the MD was assessed using self-reported methods, which can introduce recall bias and inaccuracies.

## 5. Conclusions

In conclusion, our study underscores the significant influence of adherence to the MD on health outcomes among hospitalized frail elderly patients. The interaction identified between nutritional status and MD adherence further emphasizes the importance of dietary interventions in mitigating adverse health outcomes, particularly among malnourished individuals. Encouraging adherence to the MD, especially among patients with compromised nutritional status, could be the key strategy in enhancing recovery rates and minimizing hospital stays.

Future longitudinal and multi-center studies with larger samples are needed to establish causality and further elucidate the mechanisms underlying these associations.

## Figures and Tables

**Table 1:** Baseline characteristics of the patients according to their adherence to the MD.

	High-Level Adherence (Score ≥10) n. 52 (44.4%)	Low-Level Adherence (Score < 10) n. 65 (55.6%)	*p* Value
Age, years	79.0 ± 6.6	80.0 ± 6.4	0.573
Genre F, *n* (%)	42 (80.8)	36 (55.4)	**0.004**
Comorbidities, ≥3	29 (55.8)	50 (76.9)	**0.015**
BMI, Kg/m^2^	26.0 [19.1–31.2]	29. 0 [20.1–33.0]	0.189
Hemoglobin, g/dL	11.2 ± 1.9	12.1 ± 1.3	**0.006**
WBC, *n*/mm^3^	6568 [5019–8544]	6547 [5192–7719]	0.677
Neutrophils, *n*/mm^3^	4348 [3244–5703]	4496 [3543–5722]	0.598
Lymphocytes, *n*/mm^3^	1045 [828–1481]	932 [663–1124]	0.062
Platelet, 10^3^/μL	227 [170–349]	190 [128–242]	**<0.001**
Glucose, mg/dL	117.3 ± 24.5	116.0 ± 32.5	0.820
Albumin, g/dL	3.6 ± 0.3	3.1 ± 0.6	**<0.001**
Creatinine, mg/dL	0.80 [0.67–0.80]	1.20 [1.00–2.36]	**0.015**
Total cholesterol, mg/dL	155 [129–159]	158 [122–208]	0.168
LDL, mg/dL	88 [67–109]	88 [65–137]	0.540
HDL, mg/dL	50 [45–61]	41 [35–60]	**0.040**
Triglycerides, mg/dL	74 [57–88]	125 [113–141]	**<0.001**
Ferritin, ng/mL	20 [6–308]	115 [27–817]	**0.002**
CRP, ng/mL	8.8 [6.0–14.0]	17.4 [11.7–39.1]	**<0.001**
MNA score	17.9 ± 5.8	17.0 ± 5.8	0.361
LOS, days	9 [7–11]	12 [9–18]	**<0.001**

Data are reported as mean (±SD), median [IQR], or *n* (%) as appropriate. Abbreviations: F, female; WBC, white blood cell; LDL, low-density lipoprotein; HDL, high-density lipoprotein; MNA, mini nutritional assessment; LOS, length of stay. *p* value < 0.05 were considered statistically significant (in bold).

**Table 2:** Descriptive statistics for inflammatory status and length of stay by nutritional status and adherence to the MD.

**Inflammatory Status**				
	**Adherence to MD**	***n*** **(%)**	**Mean**	**Standard Deviation**
MNA < 17	Low	30 (25.6)	3.44	±0.5
MNA < 17	High	17 (14.5)	2.35	±0.5
MNA ≥ 17	Low	35 (29.9)	2.49	±0.5
MNA ≥ 17	High	35 (29.9)	1.97	±0.6
**Length of Stay**				
	**Adherence to MD**	***n*** **(%)**	**Mean**	**Standard Deviation**
MNA < 17	Low	30 (25.6)	2.83	±0.3
MNA < 17	High	17 (14.5)	2.23	±0.2
MNA ≥ 17	Low	35 (29.9)	2.25	±0.3
MNA ≥ 17	High	35 (29.9)	2.10	±0.3

Inflammatory status is represented by the circulating level of CRP expressed as a natural logarithm, and length of stay is also expressed as a natural logarithm. Data are expressed as frequency (*n*, %), mean, and standard deviation. Abbreviations: MNA, mini nutritional assessment; MD, Mediterranean diet.

**Table 3:** Summary of multiple regression analysis for nutritional status, inflammatory status, and length of stay with adherence to the Mediterranean diet as the moderator.

**Dependent Variable: Inflammatory Status**				
	***b***	***SEB***	***t***	***p*** **Value**
Constant	2.53 [2.02, 3.04]	0.26	9.76	<0.001
MNA	−0.02 [−0.05, 0.01]	0.01	−1.72	0.880
Adherence to the MD	1.68 [1.01, 2.36]	0.34	4.94	<0.001
MNA *x* Adherence to the MD	−0.05 [−0.09, −0.02]	0.02	−2.82	0.006
**Dependent Variable: Length of Stay**				
	***b***	***SEB***	***t***	***p*** **Value**
Constant	2.15 [2.07, 2.23]	0.04	55.5	<0.001
MNA	−0.01 [−0.02, 0.01]	0.01	−1.68	0.095
Adherence to the MD	0.35 [0.24, 0.45]	0.05	6.68	<0.001
MNA *x* Adherence to the MD	−0.05 [−0.06, −0.03]	0.01	−5.02	<0.001

Inflammatory status is represented by the circulating level of CRP expressed as a natural logarithm, and length of stay is also expressed as a natural logarithm. Abbreviations: *b*, estimated regression coefficient for the predictor in the model [95%CI]; *SEB*: standard error of the regression coefficient; *t*, significance test of the regression coefficient.

## Data Availability

The data presented in this study are available on request from the corresponding author due to ethical and privacy restrictions.

## Supplementary Materials

The following supporting information can be downloaded at: https://www.mdpi.com/article/10.3390/nu16152482/s1, Table S1. Summary of Acute Conditions Leading to Hospitalization.
