# Supplementary material for: Adherence to the Mediterranean Diet Mitigates Inflammation and Hospital Stay in Frail Elderly Patients: A Moderation Analysis
Source: Nutrients. 2024 Jul 31;16(15):2482. doi: 10.3390/nu16152482 (PMC11314230; doi:10.3390/nu16152482)
Supplement: Supplementary file 1 [file nutrients-16-02482-s001.zip › nutrients-3108703-supplementary.pdf]

SUPPLEMENTARY MATERIALS

Table S1. Summary of Acute Conditions Leading to Hospitalization

	**N. of patients**	**Percentages (%)**
Heart failure	31	26.5
Renal impairment	30	25.6
Pleural effusion	17	14.5
Cirrhosis	14	12
Respiratory failure	9	7.7
Pulmonary embolism and deep venous thrombosis	7	6.0
Ischemic or haemorrhagic ictus	5	4.3
Gastrointestinal bleeding	4	3.4

This table details the acute conditions that were present at the time of hospital admission.
